# Crystal structures of chiral 2-[bis­(2-chloro­eth­yl)amino]-1,3,2-oxaza­phospho­lidin-2-one derivatives for the absolute configuration at phospho­rus

**DOI:** 10.1107/S2056989018011349

**Published:** 2018-08-24

**Authors:** Laurence N. Rohde Jr, Matthias Zeller, John A. Jackson

**Affiliations:** a Department of Chemistry, Youngstown State University, One University Plaza, Youngstown, Ohio 44555, USA; b Department of Chemistry, Purdue University, 560 Oval Dr., W. Lafayette, IN 47907-2084, USA

**Keywords:** crystal structure, oxaza­phospho­lidinone, organo­phospho­rus, bis­(2-chloro­eth­yl)amine, nitro­gen mustard

## Abstract

‘Nitro­gen mustard’ bis­(2-chloro­eth­yl)amine derivatives (2*R*,4*S*,5*R*)- and (2*S*,4*S*,5*R*)-2-[bis­(2-chloro­eth­yl)amino]-3,4-dimethyl-5-phenyl-1,3,2-oxaza­phos­pho­lidin-2-one (**2a** and **2b**, respectively), C_14_H_21_Cl_2_N_2_O_2_P, and (2*R*,4*R*)- and (2*S*,4*R*)-2-[bis­(2-chloro­eth­yl)amino]-4-isobutyl-1,3,2-oxaza­phospho­lidin-2-one (**3a** and **3b**, respectively), C_10_H_21_Cl_2_N_2_O_2_P, were synthesized as a mixture of diastereomers through a 1:1 reaction of enanti­omerically pure chiral amino alcohols with bis­(2-chloro­eth­yl)phospho­ramidic dichloride. Flash column chromatography yielded diastereomerically pure products, as supported by ^31^P NMR. The crystal structures of **2b** and **3b** were obtained to determine their absolute configuration at phospho­rus, and ^31^P NMR chemical shift trends are proposed based on the spatial relationship of the bis­(2-chloro­eth­yl)amine moiety and the chiral substituent of the amino alcohol. Oxaza­phospho­lidinones were observed to have a more downfield ^31^P NMR chemical shift when the aforementioned substituents are in a *syn* configuration and *vice versa* for when they are *anti*.

## Abstract 2

The structures and absolute stereochemistry of two pairs of diastereomeric nitro­gen mustards related to the chemotherapeutic cyclo­phosphamide were determined to test ^31^P NMR chemical shift trends proposed based on the spatial relationship of the bis­(2-chloro­eth­yl)amine moiety and the chiral substituent of the amino alcohol.

## Chemical context   

Bis(2-chloro­eth­yl)amine moieties, also known as a ‘nitro­gen mustard’, are of inter­est due their ability to alkyl­ate DNA, which hinders the cellular growth and replication of cancer cells (Einhorn, 1985 ▸). 2-[Bis(2-chloro­eth­yl)amino]-1,3λ^2^,2-oxaza­phosphinane 2-oxide, commercially sold as cyclo­phosphamide, features such a nitro­gen mustard moiety and is registered as an FDA-approved chemotherapeutic due to its cytotoxic ability. The bioactivation mechanism of cyclo­phosphamide is well known. Hy­droxy­lation occurs on the C-4 position through cytochrome P450 type enzymes and the cyclo­phosphamide β-eliminates into acrolein and an enanti­o­meric mixture of the cytotoxic phospho­ramide mustard (Takamizawa *et al.*, 1975 ▸; Borch & Millard, 1987 ▸; Sladek, 1988 ▸). Studies support an enanti­oselective metabolism *via* the administration of enanti­omerically pure cyclo­phosphamide, as expected for an enzyme-catalyzed reaction (Cox *et al.*, 1976 ▸; Fernandes *et al.*, 2011 ▸; Castro *et al.*, 2016 ▸). Therefore, it is of pharmaceutical inter­est to be able to readily identify the absolute configuration at phospho­rus of cyclo­phosphamide and other related nitro­gen mustard derivatives.

Diastereomeric 2-[bis­(2-chloro­eth­yl)]-1,3,2-oxaza­phospho­lidin-2-ones, a five-membered ring derivative of cyclo­phosphamide, have been previously synthesized from l- and d-serine, but lacked X-ray diffraction data to determine the absolute configuration at the P atom (Foster, 1978 ▸; Jackson *et al.*, 1992 ▸). Instead, the spectroscopic trends and X-ray diffraction analysis of an l-serine-derived 2-meth­oxy-1,3,2-oxaza­phospho­lidin-2-one was applied and the absolute configuration was determined by analogy (Thompson *et al.*, 1990 ▸). It was described that oxaza­phospho­lidinones with a downfield ^31^P NMR chemical shift had a *syn* configuration with respect to the exocyclic meth­oxy group and the chiral substituent of the amino alcohol, and *vice versa* for the *anti* configuration.

Herein we report the synthesis and absolute configuration at phospho­rus of chiral 2-[bis­(2-chloro­eth­yl)amino]-1,3,2-oxaza­phospho­lidin-2-ones in attempts to support these spectroscopic trends for the analysis of future potentially chemotherapeutic analogues. Bis(2-chloro­eth­yl)amine phos­pho­ramidic dichloride was synthesized following the experimental procedure described by Friedman & Seligman (1954 ▸). Enanti­omerically pure chiral amino alcohols were purchased and used to synthesize pairs of diastereomeric oxaza­phospho­lidinones, which allowed for easy separation *via* flash column chromatography.

## Structural commentary   

**Figure 1:**
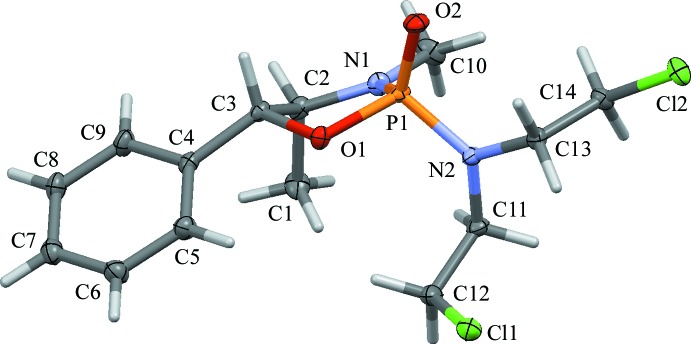
Displacement ellipsoid representation of a mol­ecule of **2b** (50% probability level), with the atom-numbering scheme.

**Figure 3:**
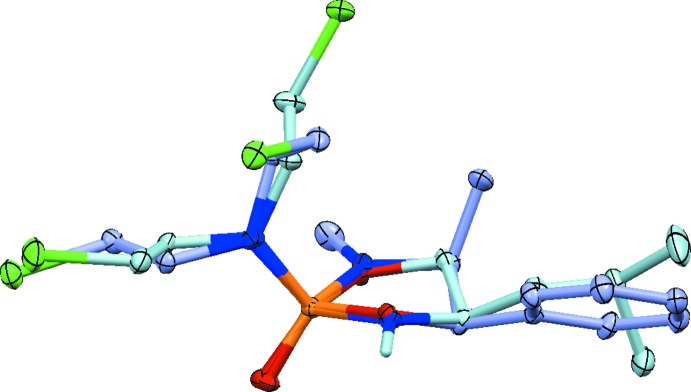
Overlay of mol­ecules **2b** and **3b** (50% displacement ellipsoid probability level). R.m.s. value based on atoms of the five-membered ring and oxygen is 0.111 Å. Color coding: P orange, O red, N blue, Cl green, and C light purple for **2b** and light blue for **3b**.

No single crystals of **3a** of X-ray diffraction quality could be obtained, and compound **2a** was isolated as an oil. Compounds **2b** and **3b**, however, have been analyzed by single-crystal diffraction (Figs. 1 ▸ and 2 ▸). The mol­ecular structures of **2b** and **3b** are similar. The five-membered rings in both structures feature the expected envelope conformation, with the flap at the C atom connecting to the phenyl and isobutyl groups, respectively. An overlay of the two structures, guided by the position of the phenyl and isobuytl groups (Fig. 3 ▸), indicates that the positions of the aza and oxo groups are swapped between **2b** and **3b**. Another slight difference between the conformations between the two rings is evident, caused by the close to planar configuration of the methyl­amine N atom of **2b** (the sum of angles around N1 is 359.97°), giving **3b** a slightly more ‘buckled’ appearance than **2b**. The chloro­ethyl moieties in **3b** are extended all-*trans*. In **2b**, one is also *trans*, while the other is *gauche* with an N2—C11—C12—Cl1 torsion angle of −65.89 (9)°.
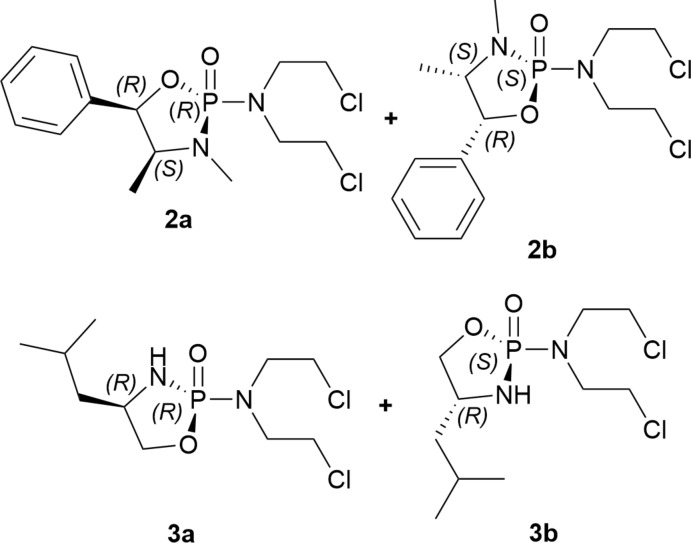


The conformation of both **2b** and **3b** appear at first sight to be stabilized by a number of weak intra­molecular hydrogen-bond-like inter­actions. In **2b**, this involves C12—H12*B*⋯O1 and C11—H11*B*⋯N1, with atoms O1 and N1 being the O and N atoms of the oxaza­phospho­lidin-2-one five-membered ring (see Table 1 ▸). In **3b**, similar inter­actions are observed for C8—H8*B*⋯O1 and C7—H7*A*⋯N1. Bond lengths and angles for these inter­actions are, however, quite unfavorable (see Table 2 ▸). In particular, atom N1 in **2b**, being essentially planar and *sp* ^2^-hybridized, appears to be an unlikely acceptor for an actual hydrogen bond. The observed close contacts are most likely not significantly contributing to the stability of the mol­ecular geometry realized in the solid state.

The absolute structure at phospho­rous has been established from the single-crystal data for both mol­ecules [Flack parameters = 0.000 (8) and 0.07 (4), respectively] to test whether their determination from ^31^P NMR chemical shift data based on the spatial relationship of the bis­(2-chloro­eth­yl)amine moiety and the chiral substituent of the amino alcohol does hold true (Thompson *et al.*, 1990 ▸). The single-crystal X-ray structures of **2b** and **3b** tentatively support the literature trends based on their ^31^P NMR chemical shifts. The chiral center(s) of the amino alcohol are *syn* to the nitro­gen mustard moiety and the absolute configurations at phospho­rus were found to both be *S* for **2b** and **3b** [see Favre & Powell (2014 ▸) for assignment of absolute structure for hypervalent atoms such as P or S in tetra­hedral geometry]. The ^31^P NMR data are shifted slightly downfield compared to their *anti* diastereomers **2a** and **3a**, thus confirming the trend proposed by Thompson *et al.* (1990 ▸). The absolute shift values are, however, rather small: 1.40 ppm for the pair of **3a** and **3b**, and nearly no shift is observed for the pair of **2a** and **2b** (0.33 ppm) (see *Experimental* section for all NMR data). Whether the assignment of absolute structure is reliable enough to be used for other related mol­ecules in the absence of structural data from X-ray diffraction is not clear based on the data at hand. For a more reliable estimate, data from a larger library of compounds are needed.

## Supra­molecular features   

**Figure 4:**
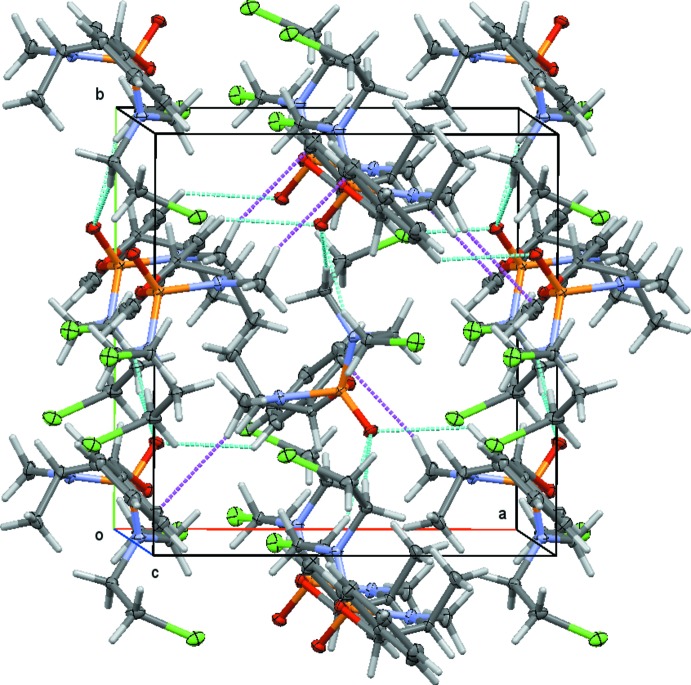
Packing arrangement and inter­molecular inter­actions of **2b** (50% probability level). Inter­molecular contacts are shown as dashed lines (light blue for C—H⋯O and purple for C—H⋯π).

Mol­ecule **2b** does not feature any acidic H atoms and, as such, does not have any strong hydrogen bonds. The O atom of the phospho­lidinone unit does, however, act as an acceptor for several C—H⋯O hydrogen-bond-like inter­actions, originating from two methyl­ene and one aromatic C—H unit of neighboring mol­ecules (see Table 1 ▸ for metrical details and symmetry operators). The three C—H⋯O inter­actions surrounding O2 are about equally spread, thus giving the O atom of the P=O unit a pseudo-tetra­hedral environment made up of the P atom on one side, and the three C—H units on the other three. A C—H⋯π inter­action, involving C10—H10*A* towards the π density of the benzene ring at (*x* − 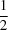, −*y* + 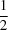, −*z* + 1), is also observed, but no significant C—H⋯Cl inter­actions and no π–π stacking are found. The combined C—H⋯O and C—H⋯π inter­actions connect mol­ecules into a three-dimensional lattice (Fig. 4 ▸).

**Figure 5:**
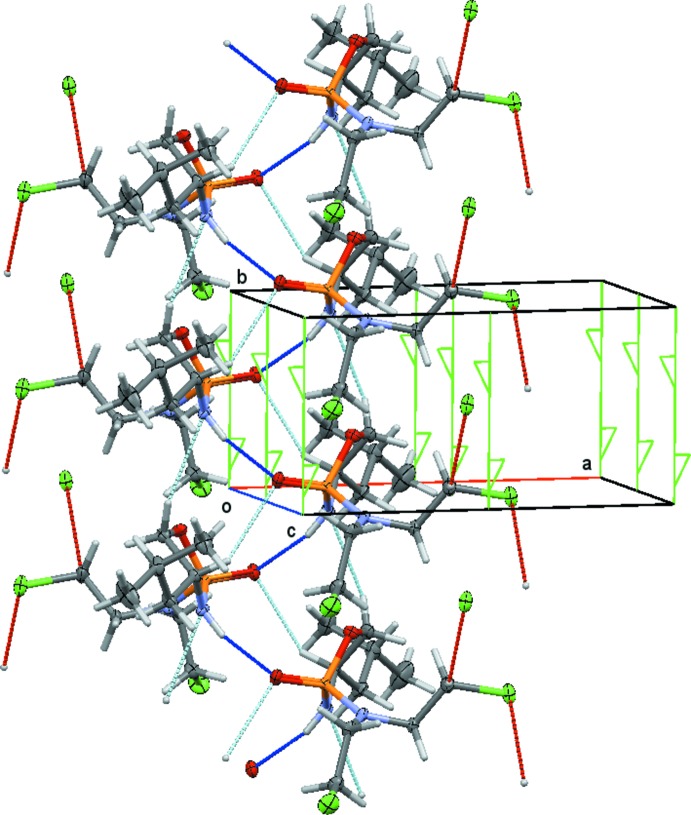
Packing arrangement and inter­molecular inter­actions of **3b** (50% probability level). Hydrogen bonds are shown as dashed lines (blue for N—H⋯O, light blue for C—H⋯O, and red for C—H⋯Cl). Mol­ecules ‘wrap’ around the twofold axis at [0, *y*, 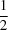] (symbolized as green lines with half arrows).

Compound **3b** does, in contrast to **2b**, have an acidic functional group, the amide N—H moiety, that is capable of forming a medium-to-strong hydrogen bond. Inter­molecular inter­actions in the structure of **3b** are indeed dominated by an N—H⋯O hydrogen bond between the amide H atom and the phospho­lidinone O atom. The graph-set motif for a single inter­action is *C*(4), connecting individual mol­ecules into infinite chains that wrap around a twofold screw axis parallel to the *b*-axis direction (Fig. 5 ▸). The spirals of mol­ecules thus formed are further stabilized by a C—H⋯O inter­action between C2 and phospho­lidinone atom O1, and by a weak C—H⋯N inter­action between atoms C1 and N1 down the chain direction (Fig. 5 ▸). Neighboring spiral chains are connected through C—H⋯Cl inter­actions involving H8*A* of one of the methyl­ene groups and Cl1.

## Database survey   

A search in the Cambridge Structural Database (Groom *et al.*, 2016 ▸) for the 2-[bis­(2-chloro­eth­yl)amino]-1,3,2-oxaza­phos­pho­l­idin-2-one fragment resulted in two entries, namely *rac*-(2*R*,5*S*)- and *rac*-(2*R*,5*R*)-2-[bis­(2-chloro­eth­yl)amino]-5-(1-napthoxymeth­yl)-1,3,2-oxaza­phospho­lidin-2-one (refcodes COKKIW and COKKES, respectively; Cates *et al.*, 1984 ▸). The single-crystal structures of COKKIW and COKKES exhibit *syn* and *trans* configurations, respectively, but unfortunately no ^31^P NMR chemical shifts have been reported to support spectroscopic trends.

## Synthesis and crystallization   

### Bis(2-chloro­eth­yl)phospho­ramidic dichloride, 1   

Bis(2-chloro­eth­yl)amine hydro­chloride (3.00 g, 16.77 mmol) was suspended in freshly distilled phosphoryl chloride (10 ml, 107 mmol) in a 50 ml round-bottomed flask and heated under reflux overnight. Once all the solids were completely dissolved, excess phosphoryl chloride was distilled off to leave a dark-brown oily residue. The residue was dissolved in an excess of a mixture of petroleum ether–acetone (1:1 *v*/*v*), while in a 323 K hot water bath. The hot solution was then filtered to remove any solids and the solvent was removed *via* rotary evaporation to yield an off-white solid. The solid was recrystallized using a 1:1 (*v*/*v*) solution of petroleum ether–acetone to afford phospho­ramide mustard **1** (4.04 g, 79.4%) as an off-white crystalline solid (m.p. 327–328 K). ^31^P NMR (162 MHz, CDCl_3_): δ 17.39. ^13^C NMR (100 MHz, CDCl_3_): δ 49.48 (*d*, *J* = 4.29 Hz), 40.82 (*d*, *J* = 2.89 Hz). ^1^H NMR (400 MHz, CDCl_3_): δ 3.77–3.62 (*m*, 8H).

### (2*R*,4*S*,5*R*)- and (2*S*,4*S*,5*R*)-2[bis­(2-chloro­eth­yl)amino]-3,4-dimethyl-5-phenyl-1,3,2-oxazaphospho­lidin-2-one (2a and 2b)   

Phospho­ramide mustard **1** (0.647 g, 2.50 mmol), (1*R*,2*S*)-(−)-ephedrine (0.375 g, 2.51 mmol), toluene (20 ml) and tri­ethyl­amine (0.75 ml, 5.38 mmol) were added to a 50 ml round-bottomed flask at 275 K under an argon atmosphere. The solution was then allowed to stir and warm to room temperature overnight. The reaction mixture was vacuum filtered through 2.0 cm of Celite packed onto a fritted glass funnel and was washed with an additional 60–80 ml of di­chloro­methane. The solvent was removed *via* rotary evaporation, which yielded a viscous yellow oil. The oil was purified by flash column chromatography (110 g silica, 100% ethyl acetate, *R*_F_ = 0.50 and 0.33 in 100% ethyl acetate) and afforded oxaza­phospho­lidinones **2a** and **2b** (combined yield 0.54 g, 64.6%), based on their order of elution. Approximately 25 mg of oxaza­phospho­lidinone **2b** was dissolved in 2 ml of ethyl acetate and allowed to slowly evaporate over several days at room temperature. This yielded colorless crystals for single-crystal X-ray diffraction.

Fast diastereomer (**2a**): 0.33 g (39.5%), clear yellow oil. *R*_F_ = 0.50 in 100% ethyl acetate. [α]_*D*_^20^ = −28.1° (*c* = 0.039 g ml^−1^). ^31^P NMR (162 MHz, CDCl_3_): δ 24.30. ^13^C NMR (100 MHz, CDCl_3_): δ 136.15 (*d*, *J* = 6.49 Hz), 128.47, 128.24, 125.86, 81.57, 59.36 (*d*, *J* = 12.76 Hz), 49.65 (*d*, *J* = 4.64 Hz), 42.43, 28.46 (*d*, *J* = 5.05 Hz), 13.87. ^1^H NMR (400 MHz, CDCl_3_): δ 7.45–7.30 (*m*, 5H), 5.49 (*dd*, 1H, *J* = 6.16, 2.24 Hz), 3.78–3.38 (*m*, 10H), 2.70 (*d*, 3H, *J* = 10.28 Hz), 0.87 (*d*, 3H, *J* = 6.60 Hz).

Slow diastereomer (**2b**): 0.21 g (25.1%), white crystalline solid (m.p. 411 K). *R*_F_ = 0.33 in 100% ethyl acetate. [α]_*D*_^20^ = −47.8 (*c* = 0.032 g ml^−1^). ^31^P NMR (162 MHz, CDCl_3_): δ 24.63. ^13^C NMR (100 MHz, CDCl_3_): δ 135.87 (*d*, *J* = 10.95 Hz), 128.55, 128.17, 125.43, 78.15 (*d*, *J* = 3.85 Hz), 59.46 (*d*, *J* = 11.89 Hz), 49.50 (*d*, *J* = 5.09 Hz), 42.42, 29.36 (*d*, *J* = 5.93 Hz), 14.78 (*d*, *J* = 1.78 Hz). ^1^H NMR (400 MHz, CDCl_3_): δ 7.45–7.22 (*m*, 5 H), 5.78 (*d*, *J* = 6.56 Hz), 3.78–3.65 (*m*, 5H), 3.63–3.40 (*m*, 4H), 2.74 (*d*, *J* = 9.60 Hz), 0.78 (*d*, *J* = 6.44 Hz).

### (2*S*,4*R*)- and (2*R*,4*R*)-2-[bis­(2-chloro­eth­yl)amino]-4-iso­butyl-1,3,2-oxaza­phospho­lidin-2-one (3a and 3b)   

Phospho­ramide mustard **1** (0.258 g, 0.99 mmol), (*R*)-(−)-2-amino-4-methyl-1-penta­nol (0.130 ml, 1.01 mmol), ethyl acetate (10 ml) and tri­ethyl­amine (0.5 ml, 3.59 mmol) were added to a 50 ml round-bottomed flask at 273 K under an argon atmosphere. The solution was then allowed to stir and warm to room temperature overnight. The reaction mixture was vacuum filtered through 2.0 cm of Celite packed on a fritted glass funnel and was washed with an additional 60–80 ml of ethyl acetate. The solvent was removed *via* rotary evaporation, which yielded a viscous yellow oil. The oil was purified by flash column chromatography (60 g silica treated with 1% tri­ethyl­amine, 100% ethyl acetate, *R*_F_ = 0.29 and 0.17 in 100% ethyl acetate) to afford oxaza­phospho­lidinones **3a** and **3b** (combined yield 0.22 g, 72.8%), based on their order of elution. Approximately 25 mg of oxaza­phospho­lidinone **3b** was dissolved in 2 ml of ethyl acetate and allowed to slowly evaporate over several days at room temperature. This yielded colorless crystals for single-crystal X-ray diffraction.

Fast diastereomer (**3a**): 0.11 g (36.4%), white crystalline solid (m.p. 371–373 °C). *R*_F_ = 0.29 in 100% ethyl acetate. [α]_*D*_^20^ = −11.1° (*c* = 0.028 g ml^−1^). ^31^P NMR (162 MHz, CDCl_3_): δ 27.58. ^13^C NMR (100 MHz, CDCl_3_): δ 71.28 (*d*, *J* = 1.85 Hz), 53.35 (*d*, *J* = 8.61 Hz), 49.12 (*d*, *J* = 5.00 Hz), 44.36 (*d*, *J* = 4.77 Hz), 42.39, 25.31, 22.93, 22.15. ^1^H NMR (400 MHz, CDCl_3_): δ 4.21 (*ddd*, 1H, *J* = 17.42 Hz, 8.77 Hz, 6.83 Hz), 3.86 (*ddd*, 1H, *J* = 8.14 Hz, 8.14 Hz, 4.40 Hz), 3.73–3.62 (*m*, 1H), 3.62–3.50 (*m*, 4H), 3.44–3.24 (*m*, 4H), 2.70 (*d*, 1H, 14.57 Hz), 1.63–1.45 (*m*, 2H), 1.39–1.29 (*m*, 1H), 0.88 (*d*, 3H, *J* = 7.16 Hz), 0.86 (*d*, 3H, *J* = 7.16 Hz).

Slow diastereomer (**3b**): 0.11 g (36.4%), white crystalline solid (m.p. 352–353 °C). *R*_F_ = 0.17 in 100% ethyl acetate. [α]_*D*_^20^ = +4.1° (*c* = 0.028 g ml^−1^). ^31^P NMR (162 MHz, CDCl_3_): δ 28.98. ^13^C NMR (100 MHz, CDCl_3_): δ 71.81, 51.30 (*d*, *J* = 9.47 Hz), 49.21 (*d*, *J* = 4.78 Hz), 44.74 (*d*, *J* = 8.80 Hz), 42.28, 25.25, 23.08, 22.04. ^1^H NMR (400 MHz, CDCl_3_): δ 4.45 (*ddd*, 1H, *J* = 11.84 Hz, 8.52 Hz, 7.09 Hz), 4.00–3.90 (*m*, 1H), 3.74 (*ddd*, 1H, *J* = 8.17 Hz, 8.17 Hz, 8.17 Hz), 3.71–3.59 (*m*, 4H), 3.56–3.35 (*m*, 4H), 2.75 (*d*, 1H, *J* = 10.92 Hz), 1.71–1.58 (*m*, 1H), 1.53–1.43 (*m*, 1H), 1.38–1.29 (*m*, 1H), 0.99 (*d*, 3H, *J* = 6.60 Hz), 0.95 (*d*, 3H, *J* = 6.56 Hz).

## Refinement   

H atoms attached to C and N atoms were positioned geometrically and constrained to ride on their parent atoms. C—H bond lengths were constrained to 0.95 Å for aromatic C—H groups. Aliphatic CH, CH_2_, and CH_3_ groups were constrained to C—H bond lengths of 1.00, 0.99, and 0.98 Å, respectively. The position of the amino H atom was refined and the N—H distance restrained to 0.88 (2) Å. Methyl H atoms were allowed to rotate, but not to tip, to best fit the experimental electron density. *U*_iso_(H) values were set to a multiple of *U*_eq_(C), with 1.5 for CH_3_ and 1.2 for N—H, C—H, and CH_2_ units. Crystal data, data collection and structure refinement details are summarized in Table 3 ▸.

## Supplementary Material

Crystal structure: contains datablock(s) 2b, 3b, global. DOI: 10.1107/S2056989018011349/fy2130sup1.cif

Structure factors: contains datablock(s) 2b. DOI: 10.1107/S2056989018011349/fy21302bsup2.hkl

Structure factors: contains datablock(s) 3b. DOI: 10.1107/S2056989018011349/fy21303bsup3.hkl

CCDC references: 1861038, 1861037

Additional supporting information:  crystallographic information; 3D view; checkCIF report

## Figures and Tables

**Figure 2:**
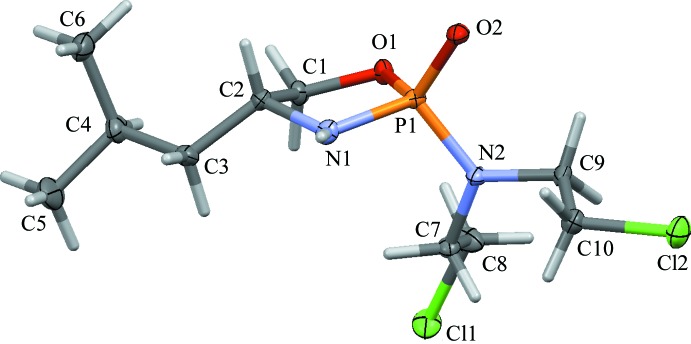
Displacement ellipsoid representation of a mol­ecule of **3b** (50% probability level), with the atom-numbering scheme.

**Table 1:** Hydrogen-bond geometry (Å, °) for **2b** [Chem scheme1]

*D*—H⋯*A*	*D*—H	H⋯*A*	*D*⋯*A*	*D*—H⋯*A*
C11—H11*A*⋯O2^i^	0.99	2.38	3.3571 (11)	170
C14—H14*B*⋯O2^i^	0.99	2.41	3.3244 (12)	153
C9—H9⋯O2^ii^	0.95	2.65	3.3444 (13)	130
C11—H11*B*⋯N1	0.99	2.63	3.1322 (11)	111
C12—H12*B*⋯O1	0.99	2.64	3.3381 (11)	128
C13—H13*A*⋯Cl1	0.99	2.86	3.4970 (9)	123
C10—H10*A*⋯C5^ii^	0.98	2.84	3.7839 (15)	162

Symmetry codes: (i) 
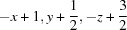
; (ii) 
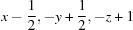
.

**Table 2:** Hydrogen-bond geometry (Å, °) for **3b** [Chem scheme1]

*D*—H⋯*A*	*D*—H	H⋯*A*	*D*⋯*A*	*D*—H⋯*A*
N1—H1⋯O2^i^	0.85 (2)	2.05 (3)	2.863 (3)	158 (4)
C2—H2⋯O2^ii^	1.00	2.57	3.401 (4)	141
C1—H1*B*⋯N1^iii^	0.99	2.71	3.481 (4)	135
C8—H8*A*⋯Cl1^iv^	0.99	2.92	3.656 (4)	132
C8—H8*B*⋯O1	0.99	2.54	3.245 (4)	128
C7—H7*A*⋯N1	0.99	2.62	3.125 (4)	112

Symmetry codes: (i) 
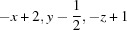
; (ii) 
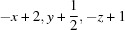
; (iii) 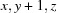; (iv) 
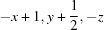
.

**Table 3:** Experimental details

	**2b**	**3b**
Crystal data		
Chemical formula	C_14_H_21_Cl_2_N_2_O_2_P	C_10_H_21_Cl_2_N_2_O_2_P
*M*_r_	351.20	303.16
Crystal system, space group	Orthorhombic, *P*2_1_2_1_2_1_	Monoclinic, *P*2_1_
Temperature (K)	100	100
*a*, *b*, *c* (Å)	10.6894 (6), 11.1623 (6), 14.0025 (7)	12.1044 (17), 5.3162 (8), 12.8933 (17)
α, β, γ (°)	90, 90, 90	90, 115.409 (4), 90
*V* (Å^3^)	1670.75 (15)	749.42 (18)
*Z*	4	2
Radiation type	Mo *K*α	Mo *K*α
μ (mm^−1^)	0.49	0.53
Crystal size (mm)	0.45 × 0.45 × 0.26	0.22 × 0.02 × 0.02

Data collection		
Diffractometer	Bruker AXS D8 Quest CMOS	Bruker AXS D8 Quest CMOS
Absorption correction	Multi-scan (*APEX3*; Bruker, 2016 ▸)	Multi-scan (*APEX3*; Bruker, 2016 ▸)
*T*_min_, *T*_max_	0.647, 0.748	0.616, 0.725
No. of measured, independent and observed [*I* > 2σ(*I*)] reflections	54792, 10531, 9765	18357, 4294, 3325
*R*_int_	0.033	0.080
(sin θ/λ)_max_ (Å^−1^)	0.910	0.716

Refinement		
*R*[*F* ^2^ > 2σ(*F* ^2^)], *wR*(*F* ^2^), *S*	0.024, 0.064, 1.07	0.048, 0.095, 1.02
No. of reflections	10531	4294
No. of parameters	193	159
No. of restraints	0	2
H-atom treatment	H-atom parameters constrained	H atoms treated by a mixture of independent and constrained refinement
Δρ_max_, Δρ_min_ (e Å^−3^)	0.39, −0.34	0.38, −0.49
Absolute structure	Flack *x* determined using 4150 quotients [(*I* ^+^) − (*I* ^−^)]/[(*I* ^+^) + (*I* ^−^)] (Parsons *et al.*, 2013 ▸)	Flack *x* determined using 1199 quotients [(*I* ^+^) − (*I* ^−^)]/[(*I* ^+^) + (*I* ^−^)] (Parsons *et al.*, 2013 ▸)
Absolute structure parameter	0.000 (8)	0.07 (4)

Computer programs: *APEX3* and *SAINT* (Bruker, 2016 ▸), *SHELXS97* (Sheldrick, 2008 ▸), *SHELXL2018* (Sheldrick, 2015 ▸), *shelXle* (Hübschle *et al.*, 2011 ▸), *Mercury* (Macrae *et al.*, 2008 ▸), and *publCIF* (Westrip, 2010 ▸).

## supplementary crystallographic information

### (2S,4S,5R)-2-[Bis(2-chloroethyl)amino]-3,4-dimethyl-5-phenyl-1,3,2-oxazaphospholidin-2-one (2b) Crystal data

**Table:**

C_14_H_21_Cl_2_N_2_O_2_P	*D*_x_ = 1.396 Mg m^−^^3^
*M**_r_* = 351.20	Mo *K*α radiation, λ = 0.71073 Å
Orthorhombic, *P*2_1_2_1_2_1_	Cell parameters from 9357 reflections
*a* = 10.6894 (6) Å	θ = 2.4–40.2°
*b* = 11.1623 (6) Å	µ = 0.49 mm^−^^1^
*c* = 14.0025 (7) Å	*T* = 100 K
*V* = 1670.75 (15) Å^3^	Block, colourless
*Z* = 4	0.45 × 0.45 × 0.26 mm
*F*(000) = 736	

### (2S,4S,5R)-2-[Bis(2-chloroethyl)amino]-3,4-dimethyl-5-phenyl-1,3,2-oxazaphospholidin-2-one (2b) Data collection

**Table:**

Bruker AXS D8 Quest CMOS diffractometer	10531 independent reflections
Radiation source: IµS microsource X-ray tube	9765 reflections with *I* > 2σ(*I*)
Laterally graded multilayer (Goebel) mirror monochromator	*R*_int_ = 0.033
ω and phi scans	θ_max_ = 40.3°, θ_min_ = 2.3°
Absorption correction: multi-scan (APEX3; Bruker, 2016)	*h* = −19→18
*T*_min_ = 0.647, *T*_max_ = 0.748	*k* = −15→20
54792 measured reflections	*l* = −23→25

### (2S,4S,5R)-2-[Bis(2-chloroethyl)amino]-3,4-dimethyl-5-phenyl-1,3,2-oxazaphospholidin-2-one (2b) Refinement

**Table:**

Refinement on *F*^2^	Hydrogen site location: inferred from neighbouring sites
Least-squares matrix: full	H-atom parameters constrained
*R*[*F*^2^ > 2σ(*F*^2^)] = 0.024	*w* = 1/[σ^2^(*F*_o_^2^) + (0.0343*P*)^2^ + 0.1289*P*] where *P* = (*F*_o_^2^ + 2*F*_c_^2^)/3
*wR*(*F*^2^) = 0.064	(Δ/σ)_max_ = 0.002
*S* = 1.07	Δρ_max_ = 0.39 e Å^−^^3^
10531 reflections	Δρ_min_ = −0.33 e Å^−^^3^
193 parameters	Extinction correction: SHELXL2018 (Sheldrick, 2015), Fc^*^=kFc[1+0.001xFc^2^λ^3^/sin(2θ)]^-1/4^
0 restraints	Extinction coefficient: 0.0137 (13)
Primary atom site location: structure-invariant direct methods	Absolute structure: Flack *x* determined using 4150 quotients [(I+)-(I-)]/[(I+)+(I-)] (Parsons *et al.*, 2013)
Secondary atom site location: difference Fourier map	Absolute structure parameter: 0.000 (8)

### (2S,4S,5R)-2-[Bis(2-chloroethyl)amino]-3,4-dimethyl-5-phenyl-1,3,2-oxazaphospholidin-2-one (2b) Special details

**Table:**

Geometry. All esds (except the esd in the dihedral angle between two l.s. planes) are estimated using the full covariance matrix. The cell esds are taken into account individually in the estimation of esds in distances, angles and torsion angles; correlations between esds in cell parameters are only used when they are defined by crystal symmetry. An approximate (isotropic) treatment of cell esds is used for estimating esds involving l.s. planes.

### (2S,4S,5R)-2-[Bis(2-chloroethyl)amino]-3,4-dimethyl-5-phenyl-1,3,2-oxazaphospholidin-2-one (2b) Fractional atomic coordinates and isotropic or equivalent isotropic displacement parameters (Å^2^)

**Table:**

	*x*	*y*	*z*	*U*_iso_*/*U*_eq_	
Cl1	0.66219 (3)	0.73317 (2)	0.57271 (2)	0.02053 (5)	
Cl2	0.65512 (2)	0.50227 (2)	0.93609 (2)	0.02110 (5)	
P1	0.50144 (2)	0.36754 (2)	0.61478 (2)	0.01080 (4)	
O1	0.53487 (6)	0.37759 (6)	0.50302 (4)	0.01294 (10)	
O2	0.57173 (7)	0.27440 (6)	0.66701 (5)	0.01656 (11)	
N1	0.35118 (7)	0.34892 (7)	0.59734 (5)	0.01437 (11)	
N2	0.52864 (7)	0.49876 (6)	0.66359 (5)	0.01273 (11)	
C1	0.25115 (10)	0.44596 (10)	0.45684 (7)	0.02017 (16)	
H1A	0.225400	0.430884	0.390754	0.030*	
H1B	0.308943	0.514081	0.458548	0.030*	
H1C	0.177284	0.464187	0.495673	0.030*	
C2	0.31606 (8)	0.33532 (8)	0.49651 (6)	0.01417 (13)	
H2	0.259710	0.264373	0.489379	0.017*	
C3	0.44360 (8)	0.30792 (7)	0.44925 (6)	0.01272 (12)	
H3	0.462543	0.220984	0.458637	0.015*	
C4	0.45200 (8)	0.33538 (7)	0.34439 (6)	0.01301 (12)	
C5	0.53407 (9)	0.41996 (9)	0.30689 (6)	0.01709 (14)	
H5	0.586804	0.464763	0.348205	0.021*	
C6	0.53872 (10)	0.43884 (9)	0.20819 (7)	0.02021 (16)	
H6	0.595012	0.496426	0.182717	0.024*	
C7	0.46181 (10)	0.37419 (9)	0.14708 (7)	0.01941 (15)	
H7	0.466005	0.386924	0.080071	0.023*	
C8	0.37859 (10)	0.29068 (10)	0.18454 (7)	0.02021 (16)	
H8	0.325004	0.246904	0.143165	0.024*	
C9	0.37388 (10)	0.27126 (9)	0.28280 (6)	0.01847 (15)	
H9	0.317110	0.213987	0.308112	0.022*	
C10	0.25759 (9)	0.34159 (10)	0.67220 (7)	0.02082 (16)	
H10A	0.215107	0.263908	0.668721	0.031*	
H10B	0.196269	0.406006	0.663841	0.031*	
H10C	0.298193	0.349897	0.734603	0.031*	
C11	0.45791 (9)	0.60577 (7)	0.63576 (6)	0.01520 (13)	
H11A	0.456531	0.662222	0.690283	0.018*	
H11B	0.370414	0.582168	0.622219	0.018*	
C12	0.51026 (11)	0.67006 (8)	0.54911 (6)	0.01917 (16)	
H12A	0.452137	0.734821	0.530021	0.023*	
H12B	0.516626	0.612916	0.495255	0.023*	
C13	0.61622 (8)	0.50952 (8)	0.74403 (5)	0.01305 (12)	
H13A	0.659371	0.587905	0.740990	0.016*	
H13B	0.680260	0.445669	0.739828	0.016*	
C14	0.54620 (8)	0.49888 (8)	0.83801 (6)	0.01539 (13)	
H14A	0.498508	0.422870	0.839319	0.018*	
H14B	0.486094	0.565913	0.844218	0.018*	

### (2S,4S,5R)-2-[Bis(2-chloroethyl)amino]-3,4-dimethyl-5-phenyl-1,3,2-oxazaphospholidin-2-one (2b) Atomic displacement parameters (Å^2^)

**Table:**

	*U*^11^	*U*^22^	*U*^33^	*U*^12^	*U*^13^	*U*^23^
Cl1	0.02609 (11)	0.01820 (9)	0.01729 (8)	−0.00405 (8)	0.00246 (7)	0.00094 (6)
Cl2	0.02295 (10)	0.02672 (10)	0.01361 (7)	0.00098 (8)	−0.00269 (7)	0.00185 (7)
P1	0.00966 (8)	0.00914 (7)	0.01360 (7)	0.00016 (6)	−0.00134 (6)	0.00005 (5)
O1	0.0097 (2)	0.0153 (2)	0.0137 (2)	−0.00138 (19)	−0.00173 (18)	−0.00211 (19)
O2	0.0174 (3)	0.0118 (2)	0.0204 (3)	0.0030 (2)	−0.0043 (2)	0.0020 (2)
N1	0.0103 (3)	0.0169 (3)	0.0159 (2)	−0.0027 (2)	−0.0004 (2)	−0.0005 (2)
N2	0.0141 (3)	0.0098 (2)	0.0143 (2)	0.0002 (2)	−0.00341 (19)	−0.0005 (2)
C1	0.0161 (4)	0.0239 (4)	0.0205 (3)	0.0075 (3)	−0.0043 (3)	−0.0026 (3)
C2	0.0101 (3)	0.0151 (3)	0.0172 (3)	−0.0010 (2)	−0.0021 (2)	−0.0024 (2)
C3	0.0111 (3)	0.0115 (3)	0.0155 (3)	−0.0003 (2)	−0.0021 (2)	−0.0022 (2)
C4	0.0116 (3)	0.0121 (3)	0.0154 (3)	0.0009 (2)	−0.0019 (2)	−0.0030 (2)
C5	0.0164 (3)	0.0173 (3)	0.0176 (3)	−0.0033 (3)	−0.0037 (3)	0.0002 (3)
C6	0.0214 (4)	0.0211 (4)	0.0182 (3)	−0.0034 (3)	−0.0024 (3)	0.0024 (3)
C7	0.0212 (4)	0.0211 (4)	0.0160 (3)	0.0028 (3)	−0.0027 (3)	−0.0014 (3)
C8	0.0205 (4)	0.0230 (4)	0.0171 (3)	−0.0019 (3)	−0.0035 (3)	−0.0060 (3)
C9	0.0187 (4)	0.0187 (4)	0.0180 (3)	−0.0044 (3)	−0.0017 (3)	−0.0052 (3)
C10	0.0135 (4)	0.0277 (4)	0.0212 (4)	−0.0014 (3)	0.0030 (3)	0.0055 (3)
C11	0.0174 (4)	0.0105 (3)	0.0178 (3)	0.0015 (3)	−0.0019 (3)	0.0000 (2)
C12	0.0277 (5)	0.0130 (3)	0.0168 (3)	−0.0013 (3)	−0.0048 (3)	0.0016 (2)
C13	0.0109 (3)	0.0148 (3)	0.0134 (2)	−0.0013 (2)	−0.0005 (2)	−0.0007 (2)
C14	0.0143 (3)	0.0174 (3)	0.0145 (3)	−0.0005 (3)	0.0005 (2)	0.0024 (3)

### (2S,4S,5R)-2-[Bis(2-chloroethyl)amino]-3,4-dimethyl-5-phenyl-1,3,2-oxazaphospholidin-2-one (2b) Geometric parameters (Å, º)

**Table:**

Cl1—C12	1.8008 (11)	C5—H5	0.9500
Cl2—C14	1.8008 (9)	C6—C7	1.3888 (14)
P1—O2	1.4765 (7)	C6—H6	0.9500
P1—O1	1.6092 (7)	C7—C8	1.3912 (15)
P1—N1	1.6378 (8)	C7—H7	0.9500
P1—N2	1.6423 (7)	C8—C9	1.3938 (13)
O1—C3	1.4573 (10)	C8—H8	0.9500
N1—C10	1.4514 (12)	C9—H9	0.9500
N1—C2	1.4688 (11)	C10—H10A	0.9800
N2—C11	1.4664 (11)	C10—H10B	0.9800
N2—C13	1.4696 (10)	C10—H10C	0.9800
C1—C2	1.5216 (13)	C11—C12	1.5167 (13)
C1—H1A	0.9800	C11—H11A	0.9900
C1—H1B	0.9800	C11—H11B	0.9900
C1—H1C	0.9800	C12—H12A	0.9900
C2—C3	1.5460 (12)	C12—H12B	0.9900
C2—H2	1.0000	C13—C14	1.5186 (11)
C3—C4	1.5026 (12)	C13—H13A	0.9900
C3—H3	1.0000	C13—H13B	0.9900
C4—C5	1.3917 (13)	C14—H14A	0.9900
C4—C9	1.3976 (12)	C14—H14B	0.9900
C5—C6	1.3989 (13)		
			
O2—P1—O1	114.69 (4)	C6—C7—C8	119.63 (9)
O2—P1—N1	118.92 (4)	C6—C7—H7	120.2
O1—P1—N1	94.68 (4)	C8—C7—H7	120.2
O2—P1—N2	109.38 (4)	C7—C8—C9	119.97 (9)
O1—P1—N2	107.65 (4)	C7—C8—H8	120.0
N1—P1—N2	110.42 (4)	C9—C8—H8	120.0
C3—O1—P1	108.45 (5)	C8—C9—C4	120.52 (9)
C10—N1—C2	120.82 (7)	C8—C9—H9	119.7
C10—N1—P1	125.13 (6)	C4—C9—H9	119.7
C2—N1—P1	114.02 (6)	N1—C10—H10A	109.5
C11—N2—C13	117.75 (7)	N1—C10—H10B	109.5
C11—N2—P1	121.64 (6)	H10A—C10—H10B	109.5
C13—N2—P1	120.31 (6)	N1—C10—H10C	109.5
C2—C1—H1A	109.5	H10A—C10—H10C	109.5
C2—C1—H1B	109.5	H10B—C10—H10C	109.5
H1A—C1—H1B	109.5	N2—C11—C12	114.06 (8)
C2—C1—H1C	109.5	N2—C11—H11A	108.7
H1A—C1—H1C	109.5	C12—C11—H11A	108.7
H1B—C1—H1C	109.5	N2—C11—H11B	108.7
N1—C2—C1	112.56 (7)	C12—C11—H11B	108.7
N1—C2—C3	101.92 (7)	H11A—C11—H11B	107.6
C1—C2—C3	113.98 (8)	C11—C12—Cl1	111.78 (6)
N1—C2—H2	109.4	C11—C12—H12A	109.3
C1—C2—H2	109.4	Cl1—C12—H12A	109.3
C3—C2—H2	109.4	C11—C12—H12B	109.3
O1—C3—C4	110.85 (7)	Cl1—C12—H12B	109.3
O1—C3—C2	105.29 (6)	H12A—C12—H12B	107.9
C4—C3—C2	115.51 (7)	N2—C13—C14	110.11 (7)
O1—C3—H3	108.3	N2—C13—H13A	109.6
C4—C3—H3	108.3	C14—C13—H13A	109.6
C2—C3—H3	108.3	N2—C13—H13B	109.6
C5—C4—C9	119.42 (8)	C14—C13—H13B	109.6
C5—C4—C3	123.01 (7)	H13A—C13—H13B	108.2
C9—C4—C3	117.56 (8)	C13—C14—Cl2	109.91 (6)
C4—C5—C6	119.82 (8)	C13—C14—H14A	109.7
C4—C5—H5	120.1	Cl2—C14—H14A	109.7
C6—C5—H5	120.1	C13—C14—H14B	109.7
C7—C6—C5	120.63 (9)	Cl2—C14—H14B	109.7
C7—C6—H6	119.7	H14A—C14—H14B	108.2
C5—C6—H6	119.7		
			
O2—P1—O1—C3	95.90 (6)	C1—C2—C3—O1	86.98 (8)
N1—P1—O1—C3	−28.97 (6)	N1—C2—C3—C4	−157.19 (7)
N2—P1—O1—C3	−142.13 (5)	C1—C2—C3—C4	−35.65 (10)
O2—P1—N1—C10	63.01 (9)	O1—C3—C4—C5	−2.20 (12)
O1—P1—N1—C10	−175.38 (8)	C2—C3—C4—C5	117.42 (9)
N2—P1—N1—C10	−64.58 (9)	O1—C3—C4—C9	177.17 (8)
O2—P1—N1—C2	−114.89 (6)	C2—C3—C4—C9	−63.20 (10)
O1—P1—N1—C2	6.71 (7)	C9—C4—C5—C6	−0.81 (14)
N2—P1—N1—C2	117.51 (6)	C3—C4—C5—C6	178.55 (9)
O2—P1—N2—C11	−171.12 (7)	C4—C5—C6—C7	0.21 (16)
O1—P1—N2—C11	63.67 (8)	C5—C6—C7—C8	0.59 (16)
N1—P1—N2—C11	−38.44 (8)	C6—C7—C8—C9	−0.79 (16)
O2—P1—N2—C13	2.45 (8)	C7—C8—C9—C4	0.19 (16)
O1—P1—N2—C13	−122.76 (6)	C5—C4—C9—C8	0.62 (15)
N1—P1—N2—C13	135.12 (6)	C3—C4—C9—C8	−178.78 (9)
C10—N1—C2—C1	75.29 (11)	C13—N2—C11—C12	100.67 (9)
P1—N1—C2—C1	−106.71 (8)	P1—N2—C11—C12	−85.61 (9)
C10—N1—C2—C3	−162.20 (8)	N2—C11—C12—Cl1	−65.89 (9)
P1—N1—C2—C3	15.81 (8)	C11—N2—C13—C14	81.68 (9)
P1—O1—C3—C4	167.41 (6)	P1—N2—C13—C14	−92.13 (8)
P1—O1—C3—C2	41.83 (7)	N2—C13—C14—Cl2	176.25 (6)
N1—C2—C3—O1	−34.55 (8)		

### (2S,4S,5R)-2-[Bis(2-chloroethyl)amino]-3,4-dimethyl-5-phenyl-1,3,2-oxazaphospholidin-2-one (2b) Hydrogen-bond geometry (Å, º)

**Table:**

*D*—H···*A*	*D*—H	H···*A*	*D*···*A*	*D*—H···*A*
C11—H11*A*···O2^i^	0.99	2.38	3.3571 (11)	170
C14—H14*B*···O2^i^	0.99	2.41	3.3244 (12)	153
C9—H9···O2^ii^	0.95	2.65	3.3444 (13)	130
C11—H11*B*···N1	0.99	2.63	3.1322 (11)	111
C12—H12*B*···O1	0.99	2.64	3.3381 (11)	128
C13—H13*A*···Cl1	0.99	2.86	3.4970 (9)	123
C10—H10*A*···C5^ii^	0.98	2.84	3.7839 (15)	162

Symmetry codes: (i) −*x*+1, *y*+1/2, −*z*+3/2; (ii) *x*−1/2, −*y*+1/2, −*z*+1.

### (2S,4R)-2-[Bis(2-chloroethyl)amino]-4-isobutyl-1,3,2-oxazaphospholidin-2-one (3b) Crystal data

**Table:**

C_10_H_21_Cl_2_N_2_O_2_P	*F*(000) = 320
*M**_r_* = 303.16	*D*_x_ = 1.343 Mg m^−^^3^
Monoclinic, *P*2_1_	Mo *K*α radiation, λ = 0.71073 Å
*a* = 12.1044 (17) Å	Cell parameters from 4852 reflections
*b* = 5.3162 (8) Å	θ = 3.1–28.1°
*c* = 12.8933 (17) Å	µ = 0.53 mm^−^^1^
β = 115.409 (4)°	*T* = 100 K
*V* = 749.42 (18) Å^3^	Rod, colourless
*Z* = 2	0.22 × 0.02 × 0.02 mm

### (2S,4R)-2-[Bis(2-chloroethyl)amino]-4-isobutyl-1,3,2-oxazaphospholidin-2-one (3b) Data collection

**Table:**

Bruker AXS D8 Quest CMOS diffractometer	4294 independent reflections
Radiation source: IµS microsource X-ray tube	3325 reflections with *I* > 2σ(*I*)
Laterally graded multilayer (Goebel) mirror monochromator	*R*_int_ = 0.080
ω and phi scans	θ_max_ = 30.6°, θ_min_ = 3.1°
Absorption correction: multi-scan (APEX3; Bruker, 2016)	*h* = −17→17
*T*_min_ = 0.616, *T*_max_ = 0.725	*k* = −7→7
18357 measured reflections	*l* = −18→17

### (2S,4R)-2-[Bis(2-chloroethyl)amino]-4-isobutyl-1,3,2-oxazaphospholidin-2-one (3b) Refinement

**Table:**

Refinement on *F*^2^	Secondary atom site location: difference Fourier map
Least-squares matrix: full	Hydrogen site location: mixed
*R*[*F*^2^ > 2σ(*F*^2^)] = 0.048	H atoms treated by a mixture of independent and constrained refinement
*wR*(*F*^2^) = 0.095	*w* = 1/[σ^2^(*F*_o_^2^) + (0.0474*P*)^2^ + 0.0175*P*] where *P* = (*F*_o_^2^ + 2*F*_c_^2^)/3
*S* = 1.02	(Δ/σ)_max_ < 0.001
4294 reflections	Δρ_max_ = 0.38 e Å^−^^3^
159 parameters	Δρ_min_ = −0.49 e Å^−^^3^
2 restraints	Absolute structure: Flack *x* determined using 1199 quotients [(I+)-(I-)]/[(I+)+(I-)] (Parsons *et al.*, 2013)
Primary atom site location: structure-invariant direct methods	Absolute structure parameter: 0.07 (4)

### (2S,4R)-2-[Bis(2-chloroethyl)amino]-4-isobutyl-1,3,2-oxazaphospholidin-2-one (3b) Special details

**Table:**

Geometry. All esds (except the esd in the dihedral angle between two l.s. planes) are estimated using the full covariance matrix. The cell esds are taken into account individually in the estimation of esds in distances, angles and torsion angles; correlations between esds in cell parameters are only used when they are defined by crystal symmetry. An approximate (isotropic) treatment of cell esds is used for estimating esds involving l.s. planes.
Refinement. The position of the amine H atoms was refined and the N-H bond distance was restrained to 0.88 (2) Angstrom.

### (2S,4R)-2-[Bis(2-chloroethyl)amino]-4-isobutyl-1,3,2-oxazaphospholidin-2-one (3b) Fractional atomic coordinates and isotropic or equivalent isotropic displacement parameters (Å^2^)

**Table:**

	*x*	*y*	*z*	*U*_iso_*/*U*_eq_	
Cl1	0.42213 (6)	0.54753 (18)	0.09135 (7)	0.0229 (2)	
Cl2	0.91388 (8)	0.02057 (18)	0.04717 (8)	0.0286 (2)	
P1	0.86712 (6)	0.58725 (13)	0.35572 (7)	0.01086 (18)	
O1	0.79674 (19)	0.8438 (4)	0.3538 (2)	0.0143 (5)	
O2	0.99383 (17)	0.6243 (4)	0.37047 (19)	0.0155 (5)	
N1	0.8406 (2)	0.4528 (5)	0.4568 (2)	0.0135 (6)	
H1	0.886 (3)	0.329 (5)	0.492 (3)	0.016*	
N2	0.7888 (2)	0.4415 (5)	0.2338 (2)	0.0134 (6)	
C1	0.7379 (3)	0.8342 (6)	0.4314 (3)	0.0164 (7)	
H1A	0.650845	0.786491	0.389001	0.020*	
H1B	0.742459	1.000426	0.467617	0.020*	
C2	0.8062 (3)	0.6388 (6)	0.5215 (3)	0.0133 (7)	
H2	0.881348	0.715273	0.582736	0.016*	
C3	0.7281 (3)	0.5206 (7)	0.5755 (3)	0.0185 (7)	
H3A	0.777041	0.389806	0.630753	0.022*	
H3B	0.656971	0.436364	0.514403	0.022*	
C4	0.6812 (3)	0.7079 (7)	0.6380 (3)	0.0211 (8)	
H4	0.633747	0.841894	0.582193	0.025*	
C5	0.5947 (4)	0.5717 (11)	0.6772 (4)	0.0441 (12)	
H5A	0.525838	0.501848	0.610291	0.066*	
H5B	0.564077	0.690589	0.716789	0.066*	
H5C	0.638821	0.435127	0.729761	0.066*	
C6	0.7853 (3)	0.8330 (8)	0.7387 (3)	0.0283 (9)	
H6A	0.838643	0.921393	0.711137	0.042*	
H6B	0.832701	0.704614	0.794716	0.042*	
H6C	0.751796	0.953468	0.775185	0.042*	
C7	0.6592 (3)	0.3876 (6)	0.1984 (3)	0.0146 (7)	
H7A	0.643680	0.364928	0.267210	0.018*	
H7B	0.636966	0.229454	0.153571	0.018*	
C8	0.5809 (3)	0.6018 (8)	0.1258 (3)	0.0224 (8)	
H8A	0.591595	0.615752	0.054077	0.027*	
H8B	0.607631	0.762141	0.168369	0.027*	
C9	0.8397 (3)	0.3806 (7)	0.1517 (3)	0.0157 (7)	
H9A	0.913708	0.483375	0.168799	0.019*	
H9B	0.778956	0.420611	0.072762	0.019*	
C10	0.8723 (3)	0.1042 (7)	0.1599 (3)	0.0185 (7)	
H10A	0.801388	0.001990	0.153912	0.022*	
H10B	0.941323	0.069086	0.235248	0.022*	

### (2S,4R)-2-[Bis(2-chloroethyl)amino]-4-isobutyl-1,3,2-oxazaphospholidin-2-one (3b) Atomic displacement parameters (Å^2^)

**Table:**

	*U*^11^	*U*^22^	*U*^33^	*U*^12^	*U*^13^	*U*^23^
Cl1	0.0133 (3)	0.0329 (5)	0.0192 (4)	0.0036 (3)	0.0037 (3)	0.0007 (4)
Cl2	0.0319 (4)	0.0341 (5)	0.0260 (5)	−0.0030 (4)	0.0185 (4)	−0.0116 (4)
P1	0.0099 (3)	0.0098 (4)	0.0117 (4)	0.0005 (3)	0.0035 (3)	−0.0001 (3)
O1	0.0175 (11)	0.0140 (11)	0.0137 (13)	0.0020 (9)	0.0089 (10)	0.0022 (10)
O2	0.0116 (9)	0.0205 (13)	0.0139 (12)	−0.0027 (9)	0.0050 (9)	−0.0040 (10)
N1	0.0134 (12)	0.0132 (14)	0.0121 (15)	0.0038 (10)	0.0038 (11)	0.0013 (11)
N2	0.0100 (12)	0.0171 (14)	0.0135 (15)	−0.0027 (10)	0.0055 (11)	−0.0046 (12)
C1	0.0158 (15)	0.0175 (17)	0.019 (2)	0.0001 (13)	0.0104 (14)	−0.0029 (15)
C2	0.0143 (14)	0.0146 (17)	0.0108 (17)	−0.0016 (12)	0.0052 (12)	−0.0021 (13)
C3	0.0186 (14)	0.0189 (17)	0.0208 (18)	−0.0065 (14)	0.0111 (13)	−0.0044 (15)
C4	0.0176 (16)	0.029 (2)	0.021 (2)	−0.0001 (14)	0.0125 (15)	−0.0038 (16)
C5	0.039 (2)	0.062 (3)	0.049 (3)	−0.021 (3)	0.036 (2)	−0.024 (3)
C6	0.0249 (19)	0.041 (2)	0.022 (2)	−0.0064 (16)	0.0135 (16)	−0.0125 (19)
C7	0.0108 (14)	0.0183 (17)	0.0132 (18)	−0.0011 (12)	0.0038 (13)	−0.0002 (13)
C8	0.0142 (14)	0.0251 (19)	0.0233 (19)	−0.0002 (15)	0.0036 (13)	0.0066 (17)
C9	0.0154 (15)	0.0225 (18)	0.0097 (17)	−0.0026 (13)	0.0057 (13)	−0.0044 (14)
C10	0.0204 (15)	0.0205 (18)	0.0153 (17)	−0.0021 (15)	0.0084 (13)	−0.0028 (16)

### (2S,4R)-2-[Bis(2-chloroethyl)amino]-4-isobutyl-1,3,2-oxazaphospholidin-2-one (3b) Geometric parameters (Å, º)

**Table:**

Cl1—C8	1.801 (3)	C4—C6	1.521 (5)
Cl2—C10	1.787 (3)	C4—C5	1.526 (5)
P1—O2	1.475 (2)	C4—H4	1.0000
P1—O1	1.603 (2)	C5—H5A	0.9800
P1—N1	1.634 (3)	C5—H5B	0.9800
P1—N2	1.641 (3)	C5—H5C	0.9800
O1—C1	1.456 (4)	C6—H6A	0.9800
N1—C2	1.465 (4)	C6—H6B	0.9800
N1—H1	0.85 (2)	C6—H6C	0.9800
N2—C7	1.462 (4)	C7—C8	1.520 (5)
N2—C9	1.471 (4)	C7—H7A	0.9900
C1—C2	1.513 (5)	C7—H7B	0.9900
C1—H1A	0.9900	C8—H8A	0.9900
C1—H1B	0.9900	C8—H8B	0.9900
C2—C3	1.529 (4)	C9—C10	1.513 (5)
C2—H2	1.0000	C9—H9A	0.9900
C3—C4	1.534 (5)	C9—H9B	0.9900
C3—H3A	0.9900	C10—H10A	0.9900
C3—H3B	0.9900	C10—H10B	0.9900
			
O2—P1—O1	113.87 (12)	C4—C5—H5A	109.5
O2—P1—N1	120.29 (13)	C4—C5—H5B	109.5
O1—P1—N1	95.71 (12)	H5A—C5—H5B	109.5
O2—P1—N2	109.14 (13)	C4—C5—H5C	109.5
O1—P1—N2	107.61 (13)	H5A—C5—H5C	109.5
N1—P1—N2	109.10 (14)	H5B—C5—H5C	109.5
C1—O1—P1	111.9 (2)	C4—C6—H6A	109.5
C2—N1—P1	111.1 (2)	C4—C6—H6B	109.5
C2—N1—H1	119 (3)	H6A—C6—H6B	109.5
P1—N1—H1	118 (2)	C4—C6—H6C	109.5
C7—N2—C9	117.2 (3)	H6A—C6—H6C	109.5
C7—N2—P1	119.5 (2)	H6B—C6—H6C	109.5
C9—N2—P1	123.0 (2)	N2—C7—C8	110.3 (3)
O1—C1—C2	106.6 (2)	N2—C7—H7A	109.6
O1—C1—H1A	110.4	C8—C7—H7A	109.6
C2—C1—H1A	110.4	N2—C7—H7B	109.6
O1—C1—H1B	110.4	C8—C7—H7B	109.6
C2—C1—H1B	110.4	H7A—C7—H7B	108.1
H1A—C1—H1B	108.6	C7—C8—Cl1	110.5 (2)
N1—C2—C1	102.7 (3)	C7—C8—H8A	109.6
N1—C2—C3	111.4 (3)	Cl1—C8—H8A	109.6
C1—C2—C3	113.1 (3)	C7—C8—H8B	109.6
N1—C2—H2	109.8	Cl1—C8—H8B	109.6
C1—C2—H2	109.8	H8A—C8—H8B	108.1
C3—C2—H2	109.8	N2—C9—C10	109.9 (3)
C2—C3—C4	114.4 (3)	N2—C9—H9A	109.7
C2—C3—H3A	108.7	C10—C9—H9A	109.7
C4—C3—H3A	108.7	N2—C9—H9B	109.7
C2—C3—H3B	108.7	C10—C9—H9B	109.7
C4—C3—H3B	108.7	H9A—C9—H9B	108.2
H3A—C3—H3B	107.6	C9—C10—Cl2	109.9 (2)
C6—C4—C5	111.0 (3)	C9—C10—H10A	109.7
C6—C4—C3	112.0 (3)	Cl2—C10—H10A	109.7
C5—C4—C3	109.1 (3)	C9—C10—H10B	109.7
C6—C4—H4	108.2	Cl2—C10—H10B	109.7
C5—C4—H4	108.2	H10A—C10—H10B	108.2
C3—C4—H4	108.2		
			
O2—P1—O1—C1	−130.6 (2)	P1—N1—C2—C3	154.3 (2)
N1—P1—O1—C1	−3.9 (2)	O1—C1—C2—N1	−34.2 (3)
N2—P1—O1—C1	108.2 (2)	O1—C1—C2—C3	−154.4 (3)
O2—P1—N1—C2	103.6 (2)	N1—C2—C3—C4	−176.1 (3)
O1—P1—N1—C2	−18.4 (2)	C1—C2—C3—C4	−61.0 (4)
N2—P1—N1—C2	−129.3 (2)	C2—C3—C4—C6	−62.3 (4)
O2—P1—N2—C7	−178.7 (2)	C2—C3—C4—C5	174.4 (3)
O1—P1—N2—C7	−54.7 (3)	C9—N2—C7—C8	−83.5 (4)
N1—P1—N2—C7	48.1 (3)	P1—N2—C7—C8	91.1 (3)
O2—P1—N2—C9	−4.4 (3)	N2—C7—C8—Cl1	−175.6 (2)
O1—P1—N2—C9	119.6 (3)	C7—N2—C9—C10	−82.3 (3)
N1—P1—N2—C9	−137.6 (3)	P1—N2—C9—C10	103.2 (3)
P1—O1—C1—C2	23.8 (3)	N2—C9—C10—Cl2	172.0 (2)
P1—N1—C2—C1	33.0 (3)		

### (2S,4R)-2-[Bis(2-chloroethyl)amino]-4-isobutyl-1,3,2-oxazaphospholidin-2-one (3b) Hydrogen-bond geometry (Å, º)

**Table:**

*D*—H···*A*	*D*—H	H···*A*	*D*···*A*	*D*—H···*A*
N1—H1···O2^i^	0.85 (2)	2.05 (3)	2.863 (3)	158 (4)
C2—H2···O2^ii^	1.00	2.57	3.401 (4)	141
C1—H1*B*···N1^iii^	0.99	2.71	3.481 (4)	135
C8—H8*A*···Cl1^iv^	0.99	2.92	3.656 (4)	132
C8—H8*B*···O1	0.99	2.54	3.245 (4)	128
C7—H7*A*···N1	0.99	2.62	3.125 (4)	112

Symmetry codes: (i) −*x*+2, *y*−1/2, −*z*+1; (ii) −*x*+2, *y*+1/2, −*z*+1; (iii) *x*, *y*+1, *z*; (iv) −*x*+1, *y*+1/2, −*z*.
